# Editorial: Steroid hormone receptors and cell cycle in breast cancer

**DOI:** 10.3389/fendo.2023.1196523

**Published:** 2023-04-13

**Authors:** Victoria T. Fabris, Laura Spring, Luisa A. Helguero

**Affiliations:** ^1^ Laboratorio de Carcinogénesis Hormonal, Instituto de Biología y Medicina Experimental, Buenos Aires, Argentina; ^2^ Massachusetts General Hospital and Harvard Medical School, Boston, MA, United States; ^3^ Institute of Biomedicine (IBiMED), University of Aveiro, Aveiro, Portugal

**Keywords:** breast cancer, hormone receptors, endocrine resistance, cell cycle inhibitors, crosstalk

Breast cancer is the most frequently diagnosed cancer in women with a high incidence and mortality worldwide. Nearly 70% of breast carcinomas express steroid hormone receptors and therefore patients can be treated with endocrine therapy. The treatment is principally targeted to block the estrogen receptor (ER) or estrogen synthesis (1). However, increasing evidence also suggests that the progesterone receptor (PR) is a potential target for therapy (2). In recent years, cell cycle inhibitors have been included in the clinical management of breast cancer patients in combination with antiestrogenic therapy (3). The aim of this Research Topic is to discuss the role of hormone receptors and cell cycle proteins in breast cancer and how their interplay could modulate the response to the different treatments.

Steroid hormone receptors belong to a ligand-activated nuclear receptor superfamily that includes ER, PR, androgen receptor, glucocorticoid receptor (GR), and mineralocorticoid receptor. These receptors are transcription factors that usually bind to the DNA as dimers, or form heterocomplexes that participate in the development and proliferation of the mammary gland and breast cancer (4). ER presents two isoforms, ERα and ERβ, which have differential roles regulating the transcription of different sets of genes (5). ERα is overexpressed in luminal breast cancer and is the gold standard to indicate antiestrogenic treatment, while ERβ is not generally expressed in breast cancer (6), with most studies found in the literature resorting to reintroduction, often overexpression, to show a tumor suppressor role of ERβ (7). Still, these approaches do not allow to discern between the effects mediated solely by ERβ homodimers or by ERβ/ERα heterodimers. In this Research Topic, Song et al. developed a novel estrogen-dependent model consisting of MCF7 Tet-Off ERβ cell line (8) in which ERα expression was deleted using CRISPR-Cas9. Comparing the two cell lines that express one or the other ER isoform in the same cellular context they show that in cells stimulated with estrogens, ERα promotes cell proliferation whereas ERβ inhibits the cell cycle at the G2/M stage. Interestingly, only ERβ appeared to modulate extracellular matrix organization and migration. Therefore, this novel work has contributed to showing that ERβ mediates tumor-suppressing activities independently of ERα expression.

Steroid hormones can bind and signal through their classical nuclear hormone receptors (nHR) and can activate the receptors located in the cytoplasmic membrane (either the classical hormone receptors or non-classical receptors coupled to G proteins, mHR). In this Research Topic, Renteria et al. describe the crosstalk between both types of PR receptors, nPR and mPR. Progesterone can exert its action through the nPR and bind also to the mPR which in turn can activate the nPR. Moreover, the antiprogestin mifepristone, which has shown antitumor effect in some clinical trials (9), can also mediate their action by mPR.

As mentioned above, hormone receptors can form heterocomplexes that are recruited on the DNA binding sites of several genes to regulate proliferation and progression in breast cancer (10). In this Topic, Pecci et al. discuss the role of the interaction between PR and GR in breast cancer. The analysis focused on cell line models expressing both receptors to demonstrate that PR and GR ligands could regulate the activation of the opposite receptor pathway and thus the transcription of the corresponding genes. Their contribution increases our understanding of how different hormone receptors interact with each other and with their co-regulators to impact cell pathways regulating proliferation, metastasis, and therapy resistance.

The cell cycle is controlled by the successive activation of complexes formed by a cyclin and the associated CDK that regulates the proper progression of the cell cycle phases from G0/G1 to mitosis (11). The cyclin D1/CDK4/6/RB/E2F1 pathway is one of the most activated in ER/PR positive breast cancer and likewise the CDK4/6 inhibitors have been approved in combination with endocrine therapy for the treatment of these tumors, after showing improved outcomes compared to monotherapy (3). Recent investigations by Vanhevel et al. propose combining the CDK4/6 inhibitor Palbociclib with an analog of vitamin D3 for the treatment of luminal carcinomas. This combination inhibits cell proliferation through arrest at the G1/S stage more efficiently than the single treatments in an ER-positive breast cancer experimental model. Interestingly, the combination therapy increased ROS production, which may account for the enhanced antitumoral effect observed.

Overcoming resistance to endocrine therapy is one of the challenges in breast cancer treatment. In this Topic, the review of Belachew and Sewasew explains the molecular mechanisms of antiestrogenic resistance. It includes loss of ER expression, mutations in the ligand binding domain, overexpression of ER co-activators or downregulation of co-repressors, the interaction of ER with growth factor receptors, and/or the regulation of ER by miRNAs. An altered expression of cell cycle proteins and polymorphisms in cytochrome P2D6 that suppress the metabolization of tamoxifen is also observed in resistant tumors. The authors propose combining endocrine therapy with targets of the molecular pathways involved in resistance.

The tumor’s cellular, molecular, and vascular heterogeneity pose a challenge to the effectiveness of any therapy. In this Topic, the review by de Oliveira et al. describes the main sources of heterogeneity contributing to endocrine resistance. Factors influencing the heterogeneity found in the tumor microenvironment vary in time and location within the tumor or metastatic site. The authors highlight the different types of cancer-associated fibroblasts inducing endocrine resistance in breast cancer that induce hormone-independence and antiestrogen resistance through a variety of mechanisms. They also summarize the role of tumor-associated macrophages through activation of breast cancer NFκB/STAT3 leading to ER ligand-independent phosphorylation. They point out the transcriptional heterogeneity that is being addressed using single-cell transcriptomics, and describe the most frequent driver mutated genes in metastatic disease. Finally, spatial and temporal, vascular, and cellular, heterogeneity in the tumor will determine nutrient and oxygen availabilities, which impact metabolic dependencies and endocrine resistance.

In conclusion, the complex interactions between steroid hormone receptors, spatial and cellular context, modulate the endocrine resistance pathways, are discussed in this Topic, and must be contemplated in the design of breast cancer therapies.

## Author contributions

VF and LH contributed to write the Editorial and all the authors revised, made corrections and approved the submitted version of the manuscript.
